# Effect of storage conditions on the shelf-life extension of fungus-colonized substrates based on *Metarhizium anisopliae* using modified atmosphere packaging

**DOI:** 10.1038/s41598-021-04232-5

**Published:** 2022-01-10

**Authors:** Seul-Gi Jeong, Ho Myeong Kim, Junheon Kim, Jae Su Kim, Hae Woong Park

**Affiliations:** 1 Eco-Friendly Process Technology Research Group, Technology Innovation Research Division, World Institute of Kimchi, Gwangju, 61755 Republic of Korea; 2 National Institute of Forest Science, Seoul, 02455 Republic of Korea; 3 Department of Agricultural Biology, College of Agricultural and Life Sciences, Jeonbuk National University, Jeonju, 54896 Republic of Korea; 4 Department of Agricultural Convergence Technology, Jeonbuk National University, Jeonju, 54596 Republic of Korea

**Keywords:** Applied microbiology, Fungal physiology, Fungal biology

## Abstract

*Metarhizium anisopliae* is a promising alternative to chemical pesticides against pine wilt disease caused by *Bursaphelenchus xylophilus*. Herein, we investigated the efficacy of modified atmosphere packaging (MAP) to prolong the shelf-life of the *M. anisopliae* conidia. The effects of various conditions on its stability were also examined. *M. anisopliae*-inoculated millet grains were treated in a MAP system with different packaging materials (polypropylene, PP; polyethylene terephthalate, PET; ethylene vinyl alcohol, EVOH), gas compositions (high CO_2_ atmosphere, ≈ 90%; high O_2_ atmosphere, > 95%; high N_2_ atmosphere, > 95%; 30% CO_2_ + 70% N_2_; 50% CO_2_ + 50% N_2_; 70% CO_2_ + 30% N_2_), and storage temperatures (4 and 25 °C). Results revealed EVOH film as the best for the preservation of gases at all concentrations for 28 days. MAP treatment in the high-barrier EVOH film under an atmosphere of 30% CO_2_ + 70% N_2_ achieved 80.5% viability of dried conidia (7.4% moisture content), with 44.2–64.9% viability recorded with the other treatments. Cold storage for technical concentrates formulation promoted extension of shelf-life of MAP-treated conidia. These results imply that MAP under optimized conditions could enhance the shelf-life of fungus-based biopesticides in fungus-colonized substrates formulations.

## Introduction

Pine wilt disease (PWD) is a fatal conifer disease that decolorizes and browns needles and eventually leads to death, affecting several species of pine trees (*Pinus* spp.) and resulting in serious environmental and economic losses worldwide^1,2^. Since these symptoms were first reported in North America (USA and Canada), this disease has spread further to Asia (Japan, China, Korea, and Taiwan), and, recently, Europe (Portugal and Spain)^3^. The pine wood nematode *Bursaphelenchus xylophilus* Nickle (Nematoda: Aphelenchoididae) is the only known agent of PWD and is transmitted from infected pine trees to healthy ones by the insect vectors, Japanese pine sawyer beetle, *Monochamus alternatus* Hope (Coleoptera: Cerambycidae) and *M. saltuarius* Gebler (Coleoptera: Cerambycidae), in East Asia^4–6^. To prevent the spread of PWD, it is essential to develop an effective method to control these insects.

A variety of synthetic chemical pesticides, including acetamiprid, thiacloprid, buprofezin, metham sodium, clothianidin, thiamethoxam, and fenitrothion, have been proposed to control PWD. However, these chemicals have been limited in most developed countries due to environmental hazards, unhealthful residues, and threat to non-target species^7–9^. For these reasons, biocontrol methods that incorporate natural predators, parasites, and microbial pathogens of the target pest need to be developed. One of these is fungus-based biopesticides, which have a low possibility of resistance development, high specificity and biodegradability, and no health risks, and among several entomopathogenic fungi species, *Metarhizium anisopliae* (Hypocreales: Clavicipitaceae) has been intensively studied and commercialized in at least 25 countries^10,11^.

The formulation types of biopesticides are categorized into dry and liquid. Dry formulations, such as seed dressing, granules, and wettable powders, account for more than 80% of fungal biopesticide products. Among them, fungus-colonized grains are the most used because of their advantages in conidia production and direct application to soil^10–12^. However, the lack of conidia stability during distribution is a critical obstacle to its widespread use and market competitiveness^11^. To overcome this issue, several research efforts, such as designed formulations, pre-incubation, and adjuvant, have been suggested^11,13,14^; however, constraints of high installation cost and obstacle of scaling up still limit commercial applications of these treatments. For the above reasons, modified atmosphere packaging (MAP) has emerged as a potential technology due to its beneficial effects on shelf-life^13,15,16^. MAP is the practice of changing the natural components of the atmosphere (O_2_, CO_2_, and N_2_) within a package. Although the efficacy of MAP treatment is affected by packaging material and gas composition, research has been focused on O_2_-absorbing packaging using O_2_ scavengers or CO_2_-emitting sachets^16,17^. In addition, little information exists on the application of MAP to *M. anisopliae*, despite many previous studies on other genera, such as *Beauveria*^13,15,16,18^.

An assessment of the effect of MAP on shelf-life extension of fungal conidia is necessary for its practical application. Thus, the overall goal of this study was to evaluate the efficacy of MAP treatment on the stability of technical concentrates (fungus-colonized millet grains) based on *M. anisopliae*. The impact of storage conditions, such as moisture content, packaging system, and storage temperature on the shelf-life extension of fungal conidia, was also comprehensively investigated.

## Results and discussion

### Effect of moisture content on thermotolerance of fungus-colonized substrates

The viability of *M. anisopliae* conidia cultured on millet grains with various moisture contents during heat treatment is listed in Table 2. At the baseline moisture level, less growth was observed with increasing heat exposure time. Conidial thermotolerance increased with decreasing moisture content from 14.3 to 7.4%. The technical concentrates with 7.4% and 9.0% moisture content achieved satisfactory conidial growth (+ or ++) for all time intervals at 50 °C. Dried mycotized millet grains (7.4% moisture content) were the only material with moisture levels that supported growth of the conidia. At 14.3% moisture content, *M. anisopliae* conidia did not grow at all tested temperatures. Regardless of the moisture content of fungus-colonized grains, high temperatures (70 °C) completely inhibited conidial growth. Our results are consistent with those of previous studies by Hedgecock et al.^19^ and Kim et al.^20^, indicating that dried conidia in granular formulations showed much superior thermotolerance to conidia with higher moisture content. For a proper environment for conidial thermotolerance, the fungus-colonized substrates should be kept dry after manufacturing the products.

### Changes in gas composition within different packages

**Figure 1:**
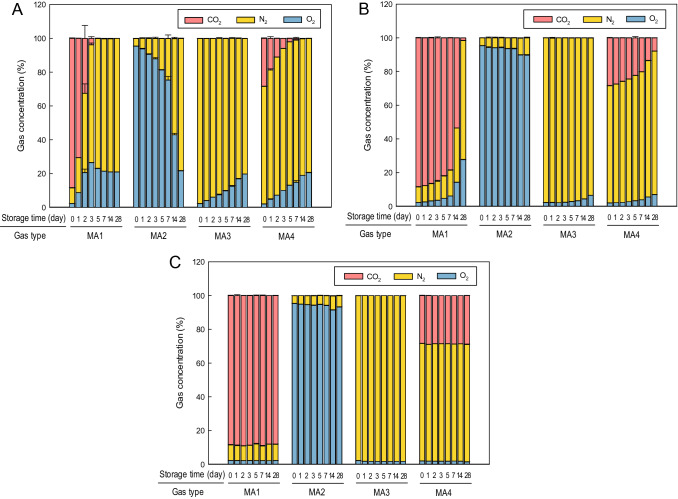
Changes in gas concentration of CO_2_, O_2_, and N_2_ in the plastic bags of polypropylene (**A**), polyethylene terephthalate (**B**), and ethylene vinyl alcohol (**C**) following treatment with modified atmosphere packaging under different gas compositions of MA1 (high CO_2_ atmosphere, ≈ 90%), MA2 (high O_2_ atmosphere, > 95%), MA3 (high N_2_ atmosphere, > 95%), and MA4 (30% CO_2_ + 70% N_2_). Error bars represent standard deviations calculated from triplicates.

Figure 1 shows the gas concentration in the plastic packaging films of polypropylene (PP), polyethylene terephthalate (PET), and ethylene vinyl alcohol (EVOH) treated with MAP. The order of sustainability of the modified atmosphere in the tested plastic films was EVOH > PET > PP. With the PP package, atmospheric equilibrium (21% O_2_ + 78% N_2_) was attained by 28 days across all MAP treatments. Statistically significant changes in CO_2_, O_2_, and N_2_ concentrations during storage were observed in the PET package, regardless of MAP treatment (*P* < 0.05), although a high CO_2_ atmosphere (MA1) was the only treatment that established the atmospheric equilibrium for 28 days. In contrast with the PP and PET packages, a steady state in the gas composition of all MAP treatments was observed in the EVOH package. These results could be attributed to the differences in the permeability of O_2_ and CO_2_ between packaging materials (Table 1). The lower permeability of O_2_ and CO_2_ caused the equilibrium arrival time to increase. Siah and Mohd Tahir^21^ and Buntinx et al.^22^ reported that multilayer sheets containing EVOH have the lowest oxygen transmission rate, followed respectively by nylon, PET, polyethylene, and PP. In short, we found that EVOH could be efficiently used as a packaging film for MAP because of its excellent barrier properties against a modified atmosphere and maintains the gas concentration within the package.

**Table 1:** Properties of plastic films used in this study^a^.

Packaging material	Abbreviation	T_m_^b^ (°C)	Density (g/cm^3^)	$P_{{O_{2} }}$^c^ (cm^3^ μm/m^2^-h-atm)	$P_{{CO_{2} }}$^d^ (cm^3^ μm/m^2^-h-atm)
Polypropylene	PP	160‒175	0.89‒0.91	1541‒2416	8368‒13,119
Polyethylene terephthalate	PET	245‒265	1.40	45	221
Ethylene vinyl alcohol	EVOH	181	1.19	0.325	10.10

^a^*Source: *Mangaraj et al. (2009).

^b^T_m_, melting temperature.

^c^$P_{{O_{2} }}$, permeability at 25 °C for O_2_.

^d^$P_{{CO_{2} }}$, permeability at 25 °C for CO_2_.

### Effect of gas composition on stability of fungus-colonized substrates

Although accelerated shelf-life testing can be used to determine the performance of fungal conidia in a relatively short time, there have been no studies on the kinetic model approach for fungal biopesticides^23^. Because of this, the thermal conditions presented in Table 2 were used to conduct subsequent experiments for accelerated shelf-life testing. Minimal thermal stress (50 °C for 2 h) was selected in accordance with the results of all samples tested as this, except for *M. anisoplia*-inoculated millet grains with moisture content of 14.3%, supported conidial growth. These conditions were also used for the thermal stress of *Cordyceps fumosorosea* conidial powder, as described previously^20^.

**Table 2:** Growth of *Metarhizium anisopliae* conidia cultured on millet with moisture contents of 7.4%, 9.0%, and 14.3% at different temperatures and treatment times.

Moisture content (%)^a^	50 °C			60 °C			70 °C		
	2 h	4 h	6 h	2 h	4 h	6 h	2 h	4 h	6 h
7.4	++ ^b^	++	+	+	‒	‒	‒	‒	‒
9.0	++	+	+	‒	‒	‒	‒	‒	‒
14.3	‒	‒	‒	‒	‒	‒	‒	‒	‒

^a^All moisture contents are expressed on a dry basis.

^b^++ , growth after incubation for 20 h at 25 °C; + , growth after incubation for 40 h at 25 °C; ‒, no growth.

The conidial germination rates of mycotized millet grains packaged in the high-barrier EVOH film following MAP treatment are shown in Table 3. The fungus-colonized substrates packaged under high O_2_ atmosphere (> 95%) had the lowest germination rate (46.3%) among all the treatments, which was lower than that under ambient atmospheric air. According to the multivariable logistic regression analysis, a rise of one percent in the oxygen concentration reduced probability of conidial germination by 0.6% (Table 4). The beneficial effects of O_2_-free environments with respect to shelf-life extension have been previously reported for some entomopathogenic fungi, including *M. anisopliae*^14,15,24^. Shelf-life extension is mainly attributed to a reduction in respiration rate, substrate uptake, oxidative reaction, and microbial metabolism, resulting in decreased production of toxic metabolites and prolonged nutrient availability^25^. Similar results were observed for mycoherbicide and nematophagous fungi^26,27^.

**Table 3:** Viability of *Metarhizium anisopliae* conidia with moisture content of 7.4% packaged in ethylene vinyl alcohol under modified atmosphere^a^.

Treatment type	Gas mixture			Germination rate (%)^b^
	CO_2_ (%)	O_2_ (%)	N_2_ (%)	
Control (w/o heat)	–	–	–	90.8 ± 0.6 a
Atmospheric air	0.03 ± 0.05 e	21.86 ± 0.22 b	78.09 ± 0.19 b	55.3 ± 1.1 d
MA1^c^	88.50 ± 0.28 a	2.25 ± 0.07 c	9.25 ± 0.21 f.	64.9 ± 3.2 c
MA2	0.05 ± 0.07 e	95.38 ± 0.02 a	4.55 ± 0.07 g	46.3 ± 3.0 e
MA3	0.05 ± 0.07 e	2.10 ± 0.14 c	97.85 ± 0.07 a	47.2 ± 2.2 e
MA4	28.40 ± 0.28 d	1.90 ± 0.14 c	69.70 ± 0.14 c	80.5 ± 2.0 b
MA5	49.01 ± 0.15 c	1.83 ± 0.07 c	49.16 ± 0.17 d	53.7 ± 2.4 d
MA6	69.12 ± 0.11 b	1.72 ± 0.09 c	29.16 ± 0.21 e	44.2 ± 1.8 e

^a^Mean ± standard deviation from three replicates. Values in the same column followed by different letters are significantly different (*P* < 0.05).

^b^The percent germination of conidia was determined by exposing it to thermal stress (50 °C for 2 h).

^c^MA1, high CO_2_ atmosphere (≈ 90%); MA2, high O_2_ atmosphere (> 95%); MA3, high N_2_ atmosphere (> 95%); MA4, 30% CO_2_ + 70% N_2_; MA5, 50% CO_2_ + 50% N_2_; MA6, 70% CO_2_ + 30% N_2_; atmospheric air, 21% O_2_ + 78% N_2_.

**Table 4:** Multivariable logistic regression analysis using the best-fit binomial generalized linear model of viability of *Metarhizium anisopliae* conidia^a^.

Stability assay	Variables	Odds ratio	95% Confidence interval	*P* value
Accelerated shelf-life testing	Gas type			
	CO_2_ (%)	1.001	1.000‒1.003	0.138
	O_2_ (%)	0.994	0.993‒0.996	< 0.001*^b^
	N_2_ (%)	NA^c^	NA	NA
	Packaging film			
	Ethylene vinyl alcohol	1		< 0.001*
	Polyethylene terephthalate	0.820	0.742‒0.906	0.002*
	Polypropylene	0.609	0.551‒0.673	< 0.001*
Actual storage testing	Storage condition			
	Temperature (°C)	0.861	0.855‒0.867	< 0.001*
	Period (month)	0.603	0.585‒0.622	< 0.001*

^a^Percentage changes in the main text was derived from the odds ratio by taking exponentials, subtracting 1, and multiplying by 100.

^b^Asterisks indicate statistically significant associations (*P* < 0.05).

^c^NA, not applicable.

Despite these benefits of O_2_ exclusion, to date, there has been no published information on replacing packaging air with CO_2_ and N_2_ in consideration of their mixing ratio for *M. anisopliae* conidia. In the present study, the viability of dried conidia (7.4% moisture content) packed in EVOH film under MA4 (30% CO_2_ + 70% N_2_) was maintained at > 80% during accelerated shelf-life testing, whereas MA5 (50% CO_2_ + 50% N_2_) and MA6 (70% CO_2_ + 30% N_2_) had significantly reduced germination rates of 53.7% (*P* < 0.05, *F*_[1,4]_ = 674.73) and 44.2% (*P* < 0.05, *F*_[1,4]_ = 30.06), respectively. Abellana et al.^25^ concluded that CO_2_ is more important in a gas mixture than N_2_ because the latter is an inert gas and has no antifungal properties. As with the relationship between CO_2_/N_2_ ratio and conidial germination rate, there was an optimal proportion identified for retaining high germination; thus, our results indicate that gas composition should be taken into account to maximize the efficacy of MAP on fungal biopesticides.

### Effect of packaging material on the stability of fungus-colonized substrates

**Figure 2:**
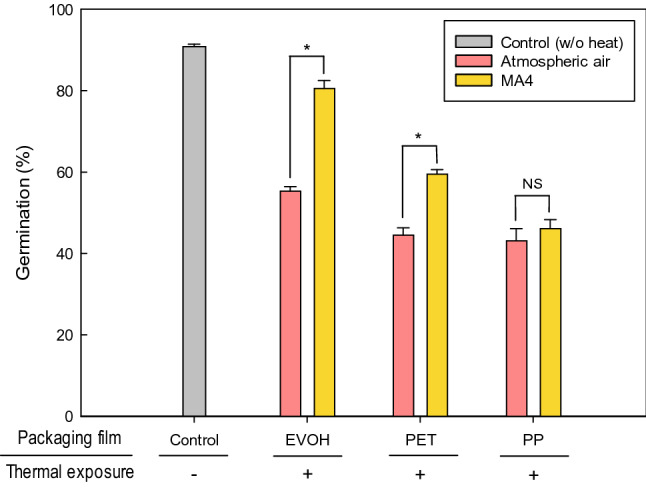
Viability of of *Metarhizium anisopliae* conidia with moisture content of 7.4% packaged in ethylene vinyl alcohol, polyethylene terephthalate, or polypropylene under atmospheric air and modified atmosphere (MA4; 30% CO_2_ + 70% N_2_) after thermal stress. Error bars represent standard deviations calculated from triplicates.

The viability of dried *M. anisopliae* conidia from mycotized millet grains packaged in different packaging materials following MAP treatment under an optimal gas mixture proportion (30% CO_2_ + 70% N_2_) is depicted in Fig. 2. Except for the PP package (*P* = 0.24, *F*_[1,4]_ = 1.98), there were significant differences in germination rates between untreated and MAP-treated technical concentrates within the EVOH (*P* < 0.05, *F*_[1,4]_ = 365.72) and PET packages (*P* < 0.05, *F*_[1,4]_ = 154.27) after thermal stress. The EVOH film are 1.22 and 1.64 times more likely to develop conidial germination than PET and PP film, respectively (Table 4). Although coextruded or laminated plastic films are widely used in the MAP industry^28^, there has been no comprehensive research on the stability of fungal conidia treated with MAP relative to plastic packaging films. However, other researchers have conducted studies on the effect of packaging material on yeast survival. Carbó et al.^29^ reported that the biocontrol agent *Candida sake* CPA-1 packaged with aluminum-based bags had higher viability than those packed in transparent plastic bags due to their permeability (0.76 cm^3^/m^2^-day and 8 cm^3^/m^2^-day, respectively). This result was similar to our data, which showed an inverse relationship between the permeability of the plastic film and conidial germination, and are also in agreement with the impact of packaging film on changes in gas composition within packages (Fig. 1), resulting from their inherent permeability.

### Effect of storage temperature on stability of fungus-colonized substrates

**Figure 3:**
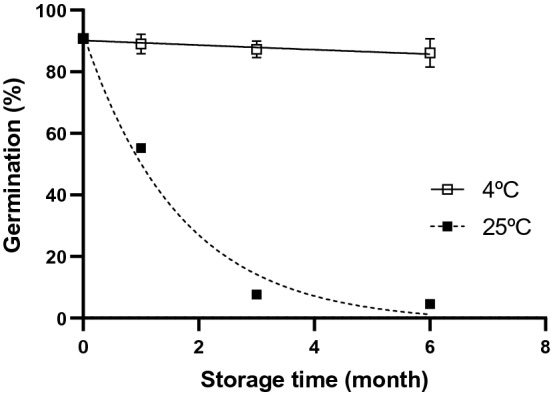
Standardized survival curves for MAP-treated *Metarhizium anisopliae* conidia under optimized conditions (7.4% moisture content, ethylene vinyl alcohol packaging, 30% CO_2_ + 70% N_2_) stored at 4 °C (□, solid line, R^2^ = 0.91) or 25 °C (■, dashed line, R^2^ = 0.98). Error bars represent standard deviations calculated from triplicates.

The storage stability of MAP-treated fungus on millet under optimized conditions (7.4% moisture content, EVOH film, 30% CO_2_ + 70% N_2_) during storage periods are presented in Fig. 3. At the same storage temperature, conidial germination decreased with increasing storage duration. In the case of storage at 25 °C, the germination rate of *M. anisopliae* conidia was dramatically reduced to 4.6% after 6 months of storage in millet. Slightly lower reductions in the germination rate were also detected when mycotized grains were stored at 4 °C; however, over the entire storage period, statistically significant differences were not observed (*P* = 0.34, *F*_[3,8]_ = 1.29). A high germination rate of 86.1% was maintained for up to 6 months in the fungus-colonized substrates at 4 °C. As the ratio of conidial germination to storage time was found to be highly correlated (R^2^ > 0.91), the half-lives, meaning the storage time required to reduce conidial germination rates by 50%, of conidia stored under different temperature were calculated. The half-life time of MAP-treated conidia was longer when stored at 4 °C than at 25 °C (69.90 months and 1.01 months, respectively). A rise of one degree and one month reduced probability of conidial germination by 13.9% and 39.7%, respectively (Table 4). The viability of biocontrol fungi during long-term storage is affected by storage temperature, humidity, conidial moisture content, strain selection, and a few other factors, although storage temperature is generally regarded as the most crucial factor^26,30,31^. Under refrigeration temperature (ca. 4 °C), metabolic activities of entomopathogenic fungi and depletion of nutrients are retarded, resulting in favorable conditions for long-term storage^32^.

## Conclusion

The present study suggests that proper MAP can be effectively used to prolong the shelf-life of *M. anisopliae* conidia cultured on millet grains. The effects of MAP were dependent on the conidial moisture content, packaging material, gas composition, and storage temperature. The results of this study are fundamental in order to understand the response of fungus-colonized millet grains to MAP treatment conditions, and by extension, to apply commercial MAP treatment. Although a high germination rate (> 80%) was achieved in both the accelerated test conditions and the actual storage, the industrial scale for extending shelf-life should be based on bioassay including virulence tests of *M. anisopliae* conidia on insect vectors of PWD in field conditions. Further studies are also needed to examine the mechanism by which MAP treatment improves storage stability. With an improved understanding of the process condition optimization, MAP could be a prospective strategy for enhancing the shelf-life of other fungus-based biopesticides.

## Methods

### Entomopathogenic fungal strain

*M. anisopliae* JEF-279 (KFCC11721P), isolated from Korean soil (Mt. Jiri, Gyeongnam, Republic of Korea), was obtained from the Department of Agricultural Biology Culture Collection at Jeonbuk National University (Jeonju, Republic of Korea)^33^. Stock cultures were kept frozen at –70 °C in 0.7 mL of quarter-strength Sabouraud dextrose broth (Difco Laboratories, Detroit, MI, USA) with 0.3 mL of 50% glycerol. Working cultures were spread onto quarter-strength Sabouraud dextrose agar (SDA; Difco Laboratories) and incubated in the dark for 10 days at 25 °C to induce conidial production. Conidia were dislodged by adding 0.05% Tween 80 (Junsei, Tokyo, Japan) to the quarter-strength SDA plate and rubbing with a sterile cotton swab.

### Sample preparation

Millet grain, *Panicum miliaceum* L. (Poales: Poaceae), was purchased from Hyundainongsan Co., Gyeonggi Province, Republic of Korea, and was used as a substrate for conidial production of *M. anisopliae* JEF-279, as described by Kim et al.^12^. Briefly, 200 g of grain was placed in a polyethylene bag, soaked in 100 mL of distilled water containing 160 μL of 50% citric acid, treated in a microwave oven for 4 min, and sterilized by autoclaving for 15 min at 121 °C. After cooling and drying inside a biosafety hood (24 ± 2 °C) with the fan running, 1 mL of conidial suspension (ca. 10^7^ conidia/mL) was inoculated into the bag and mixed thoroughly. The bag was held for 14 days at 24 ± 2 °C with a 16-h light/day photoperiod, and then the inoculated samples were dried for six days inside a desiccator (24 ± 2 °C).

To analyze the relationship between moisture content and temperature, *M. anisopliae*-inoculated millet grains were studied with the samples in bags at different moisture levels. Three moisture levels (7.4%, 9.0%, and 14.3%) of technical concentrates were selected, considering the possible moisture range where millet grains will be in equilibrium or not become sticky. The original moisture content of the mycotized millet grains was 9.0%, which was determined using a halogen moisture analyzer (MB45; Ohaus, New Jersey, USA). To reduce moisture level up to 7.4%, moisture absorber (silica gel) were added to fungus-colonized substrates and dried overnight. For 14.3% moisture content, predetermined amounts of sterile distilled water were added to each sample in polyethylene bags and mixed by hand for 10 min to ensure even distribution of moisture throughout the sample, and immediately followed by subsequent experiments for thermotolerance assay and MAP treatment.

### Thermotolerance assay

To investigate not only the impact of moisture content on the thermotolerance of fungus-colonized substrates but also establish the conditions for accelerated shelf-life testing, the growth of spores was tested on quarter-strength SDA. The mycotized millet grains with different moisture contents (as above) were placed in a convection oven (OF-22; Jeio Tech. Co. Ltd., Daejeon, Republic of Korea) at 50, 60, and 70 °C for 6 h. At an interval of two hours, heat-treated 1-g samples were instantly transferred into sterile conical tubes containing 9 mL of 0.05% Tween 80, which were pre-chilled in a 4 °C refrigerator. After agitation, 2 μL of this suspension was inoculated on SDA and incubated for up to 40 h at 25 °C, considering the possible incubation time range where typical white-opaque colonies will be observed. The amount of growth on test medium is described in a semiquantitative manner as “many” (++), “moderate” ( +), or “without” (–), depending on how fast colonies appear, where conidia that grew after 20 or 40 h would be described as “many” (++) or “moderate” ( +), respectively.

### Experimental apparatus for MAP

The MAP system consisted of a gas mixer (KM200-3MEM; WITT-Gastechnik GmbH & Co KG, Witten, Germany), a gas flush packaging machine (AZ-450E; Intrise Co. Ltd., Gyeonggi, Republic of Korea), and a gas analyzer (OXYBABY 6.0; WITT-Gastechnik GmbH & Co KG). The gas mixer for high-purity CO_2_, O_2_, and N_2_ provided infinitely diverse mixtures with a percentage scale using individual mixing valves with a maximum gas inlet pressure of 290 psi. The packaging machine connected to the gas mixer sucked in air inside the sample bags with a nozzle, flushed the desired gas mixture at a gas inlet pressure of 28 psi, and then sealed the bags with a hot-wire sealing bar. The concentrations of CO_2_, O_2_, and N_2_ in the packages were measured by inserting a needle probe connected to the gas analyzer through a rubber septum on the packaging film.

### MAP treatment

Ten grams of mycotized millet grains were prepared for subsequent MAP treatment. To optimize the conditions, the following experiments were conducted sequentially. For MAP with different packaging materials, each type of plastic film (PP, PET, and EVOH) was coextruded as an internal layer within PP, and these multilayer sheets were tailored so that the final sample bags had a constant size of 130 × 200 mm and 100 μm thickness. The technical specifications of the packaging materials are listed in Table 1. The mycotized grain samples were packaged in an optimum plastic package under six types of modified atmospheres and one representing atmospheric air. The following gas compositions were used: MA1 (high CO_2_ atmosphere, ≈ 90%), MA2 (high O_2_ atmosphere, > 95%), MA3 (high N_2_ atmosphere, > 95%), MA4 (30% CO_2_ + 70% N_2_), MA5 (50% CO_2_ + 50% N_2_), MA6 (70% CO_2_ + 30% N_2_), and atmospheric air (21% O_2_ + 78% N_2_). To examine the effect of storage temperature, fungus-colonized millet grains packed in the optimum packaging material and modified atmosphere as determined by the abovementioned experiment were individually prepared for each storage period and stored for 6 months at 4 °C and 25 °C.

### Storage stability assay

Stability tests were divided into two groups: accelerated shelf-life testing for various MAP treatment conditions and for MAP-treated samples under optimized conditions during actual storage periods. The stability of fungus-colonized substrates was assessed by measuring the conidial germination rate. Each treated sample (1 g) was immediately transferred into a sterile conical tube containing 9 mL of 0.05% Tween 80, agitated vigorously, and then diluted to 10^7^ conidia/mL using a hemocytometer (C-Chip; NanoEntek Inc., Seoul, Republic of Korea). Two-milliliter conidial suspensions were plated onto quarter-strength SDA and incubated at 25 °C for 20 h. The germination percentage was calculated by randomly counting 100 conidia using a phase-contrast microscope (BX41; Olympus, Tokyo, Japan).

All experiments in this research complied with relevant institutional, national, and international guidelines and legislation. We are adhere to national guidelines and legislation, Pesticide Control Act.

### Modeling of survival curves

The conidial germination following three repeated MAP treatment were plotted on a percentage scale against storage time. Nonlinear regression analyses (Semi-log linear model and Exponential plateau model) were conducted to determine model parameter values and regression coefficients using GraphPad Prism (GraphPad Software, San Diego, CA, USA).

The Semi-log linear model were fitted to the survival curve of conidia stored at 4 °C and its equation is described as:

$$ {\text{y}} = 10^{{\left( {\alpha x + \beta } \right)}} $$

where $\alpha$ and $\beta$ value represents the slope and y-intercept, respectively.

The Exponential plateau model were fitted to the survival curve of conidia stored at 25 °C and its equation is given by:

$$ {\text{y}} = Y_{m} - \left( {Y_{m} - Y_{0} } \right) \times e^{ - kx} $$

where $Y_{m}$ and $Y_{0}$ mean the maximum and starting germination values, respectively, and *k* value determines is the rate constant.

The regression coefficient (R^2^) was used to estimate the goodness-of-fit of the fitted model. Also, half-life was calculated by the ‘Goal seek’ function in Microsoft Excel 2013.

### Statistical analysis

All experiments were performed three times with duplicate samples. All data were analyzed by one-way analysis of variance using Statistical Package for the Social Sciences software (version 19.0; SPSS Inc., Chicago, IL, USA). Mean values were analyzed by Duncan’s multiple range test to determine significant differences in the treatments at *P* < 0.05. For the multivariable logistic regression analysis, the binomial general linear model were used with gas type, packaging film, storage temperature, or storage period as the variables.
